# Data on the correlations among brand value, market capitalization, and consolidated overseas sales ratios of Japanese companies

**DOI:** 10.1016/j.dib.2019.103808

**Published:** 2019-03-04

**Authors:** Hiroshi Matsumura, Tomohisa Ueda, Yoshimasa Sagane

**Affiliations:** a Department of Business, Natural Resource and Economic Development, Tokyo University of Agriculture, 196 Yasaka, Abashiri Hokkaido 099-2493, Japan; b Department of Food, Aroma and Cosmetic Chemistry, Faculty of Bioindustry, Tokyo University of Agriculture, 196 Yasaka, Abashiri Hokkaido 099-2493, Japan

## Abstract

This data article features a figure and tables that show the correlations among brand value by Brand Valuation (Interbrand Japan, Inc. Tokyo, Japan), market capitalization, and the consolidated overseas sales ratios of Japanese companies. The figure shows the scatter plot for market capitalization vs. brand value for Japanese companies. The lines in the plot show the regression fits for two groups of companies (domestic and global) with consolidated overseas sales ratios below or above 30%, respectively. The Pearson's correlation coefficients between brand value and market capitalization are calculated for global and domestic companies separately. Additionally, cross-tabulation statistics and Chi-square test of independence for brand values and consolidated overseas sales ratios were performed to assess their correlation.

**Table:** Specifications table

Subject area	*Finance*
More specific subject area	*Corporate valuation*
Type of data	*Graph and tables*
How data was acquired	*Data were acquired from the websites of Interbrand Japan, Inc. and Yahoo Japan Finance.*
Data format	*Analyzed*
Experimental factors	*Brand values and consolidated overseas sales ratios were collected from the Interbrand web site. Market capitalization values were collected from Yahoo Japan's finance website.*
Experimental features	*Scatter plots and regression lines were produced using brand value and market capitalization. The consolidated overseas sales ratios are indicated by the size of the dots in the plots. The significance of the relationship between brand value and market capitalization and consolidated overseas sales ratios were assessed using Pearson's correlation coefficients and Chi-squared test.*
Data source location	*Japan*
Data accessibility	*Data are presented within this article.*
Related research article	*N/A*

**Table:** 

**Value of the data** • The data indicate that the brand value published by a branding agency is related closely to global sales and market capitalization. • The data indicate that research on a company's brand value should include its market capitalization to characterize the company. • The data imply that researchers should consider the global sales ratios to characterize the company's brand. • The data indicate the importance of considering both the global development and brand value when investigating a company's overseas expansion.

## 1 Data

**Fig. 1:**
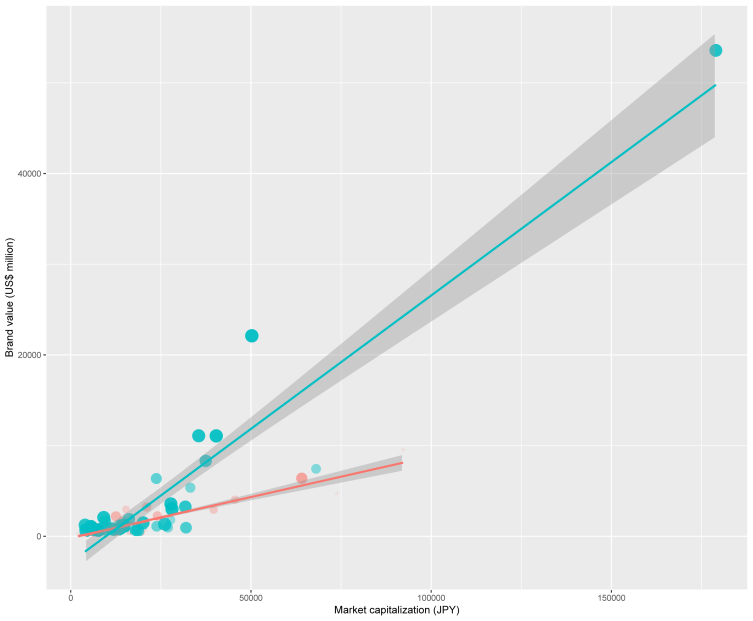
Scatter plot showing correlation between the brand values and market capitalizations of Japanese companies. Linier regression lines with 95% confidence intervals (gray). The lines were created for each group of domestic (magenta) and global (cyan) firms separately. The size of the circles indicates the consolidated overseas sales ratios.

This data article provides five sets of data: one figure and four tables. Table 1 shows brand values, consolidated overseas sales ratios, and market capitalization of 80 Japanese companies used in this article. The figure indicates the brand value (Interbrand Japan, Inc., Tokyo, Japan) of Japanese companies related to both their market capitalization and consolidated overseas sales ratios (Fig. 1). A scatter plot and regression lines indicate the correlation between brand value and market capitalization. We created regression lines for each global and domestic company with consolidated overseas sales ratios of above and below 30%. Table 2 presents the Pearson's correlation coefficients between brand value and market capitalization. The Pearson's correlation coefficients between brand value and market capitalization were higher for global and domestic companies when calculated separately compared to when they are calculated together, as Table 1 shows. The consolidated overseas sales ratio also relates to brand value. The cross-tabulation statistics and Chi-square test of independence (Table 3, Table 4) supports this finding.

**Table 1:** Brand values, consolidated overseas sales ratios and market capitalizations of eighty Japanese companies used in this data article to analyze.

Brand	Sector	Brand value (US dollar)	Oversea sales ratio	Global/Domestic^a^	Market cap (JPY)
NTT DOCOMO	Telecommunications	9,543	5.0%	Domestic	10,452,533
Softbank	Telecommunications	6,397	55.2%	Domestic**	7,266,560
Au	Telecommunications	4,718	5.0%	Domestic	8,364,618
SMFG	Financial Services	4031	24.7%	Domestic	5,165,545
Recruit	Media	3232	35.6%	Domestic**	2,385,650
Rakuten	Internet Services	2968	19.6%	Domestic	1,736,412
Mizuho	Financial Services	2929	25.2%	Domestic	4,495,915
Kao	Personal Care	2247	35.0%	Domestic**	2,724,120
Suntory	Food & Beverages	2201	41.6%	Domestic**	1,419,855
Kirin	Alcohol & Others	1666	34.6%	Domestic**	1,652,055
Asahi	Alcohol & Others	1516	13.7%	Domestic	1,813,931
Lawson	Convenience Store	1297	5.0%	Domestic	800,394
NISSIN	Fast Moving Consumer Goods	1208	21.7%	Domestic	714,179
Japan Airlines	Logistics	1123	39.0%	Domestic**	1,122,932
SECOM	Security	1017	5.0%	Domestic	1,769,028
Mitsubishi Estate	Construction & Real Estate	922	5.0%	Domestic	2,894,713
Kampo Seimei	Financial Services	795	5.0%	Domestic	1,321,200
FamilyMart	Convenience Store	740	12.6%	Domestic	833,767
Calbee	Fast Moving Consumer Goods	685	11.9%	Domestic	509,739
Mitsui Fudosan	Construction & Real Estate	675	5.0%	Domestic	2,370,001
Meiji	Food & Beverages	616	5.0%	Domestic	1,600,122
NEC	Computer Services	616	21.4%	Domestic	731,930
LINE	Internet Services	612	29.6%	Domestic	934,818
KOSE	Cosmetics	586	17.7%	Domestic	581,082
Daiwa House	Construction & Real Estate	563	5.0%	Domestic	1,922,763
NTT DATA	Business Services	556	31.2%	Domestic**	1,520,310
Dai-ichi Life	Financial Services	548	17.9%	Domestic	1,846,153
ORIX	Financial Services	476	22.9%	Domestic	2,205,220
TOTO	Construction & Real Estate	442	24.0%	Domestic	743,321
BANDAI NAMCO	Entertainment	438	25.2%	Domestic	699,300
Yamato	Transportation	424	5.0%	Domestic	985,159
Sekisui House	Construction & Real Estate	414	5.0%	Domestic	1,232,365
ABC-MART	Apparel	405	26.9%	Domestic	527,382
Resona	Financial Services	390	5.0%	Domestic	1,083,039
Sompo Holdings	Financial Services	370	12.2%	Domestic	1,413,859
Sumitomo Mitsui Trust	Financial Services	345	12.2%	Domestic	1,385,738
Gusto	Restaurants	289	5.0%	Domestic	330,121
Matsumotokiyoshi	Retail	282	5.0%	Domestic	222,369
Nitori	Retail	260	5.0%	Domestic	1,438,555
Sumitomo Realty Development	Construction & Real Estate	251	5.0%	Domestic	1,315,426
Toyota	Automotive	53,580	77.9%	Global	20,291,687
Honda	Automotive	22,106	86.1%	Global	5,695,131
Canon	Electronics	11,081	81.2%	Global	4,023,964
Nissan	Automotive	11,066	85.2%	Global	4,576,081
Sony	Electronics	8315	71.4%	Global	4,245,563
MUFG	Financial Services	7435	38.5%	Global	7,716,358
Panasonic	Electronics	6365	52.3%	Global	2,691,000
UNIQLO	Apparel	5356	42.2%	Global	3,761,372
Subaru	Automotive	3566	81.3%	Global	3,149,006
Nintendo	Electronics	3250	73.1%	Global	3,604,059
Bridgestone	Automotive	2997	82.5%	Global	3,183,296
Mazda	Automotive	2072	80.6%	Global	1,036,285
Suzuki	Automotive	1893	67.1%	Global	1,831,430
Tokio Marine	Financial Services	1808	33.4%	Global	3,142,969
Shiseido	Personal Care	1639	61.1%	Global	1,083,200
Komatsu	Machinery	1496	77.6%	Global	2,277,320
Daikin	Machinery	1340	75.4%	Global	2,954,589
Muji	Retail	1275	35.1%	Global	630,070
ASICS	Sporting Goods	1246	76.4%	Global	448,317
Unicharm	Personal Care	1205	61.1%	Global	1,551,155
Shimano	Machinery	1190	90.8%	Global	1,665,251
Ricoh	Printers, Machinery	1146	65.5%	Global	637,645
Hitachi	Diversified	1105	48.9%	Global	2,702,873
Nikon	Precision Equipment	1017	85.8%	Global	636,195
Mitsubishi Electric	Diversified	970	42.6%	Global	3,054,394
DENSO	Automotive	946	61.4%	Global	3,624,924
Yakult	Food & Beverages	931	41.1%	Global	861,960
Yamaha	Diversified	900	84.8%	Global	1,557,743
Olympus	Precision Equipment	825	79.7%	Global	1,285,018
Nomura	Financial Services	789	31.8%	Global	2,012,579
Fujitsu	Computer Services	779	40.0%	Global	1,289,828
Epson	Printers, Machinery	732	75.8%	Global	852,421
Isuzu	Automotive	723	64.0%	Global	1,102,525
Kikkoman	Food & Beverages	714	56.7%	Global	703,732
Ajinomoto	Food & Beverages	711	53.1%	Global	1,336,159
Konica Minolta	Electronics	702	80.6%	Global	473,007
Kubota	Machinery	690	67.7%	Global	2,109,516
Fujifilm	Chemical	657	59.6%	Global	2,045,637
Mitsubishi Motors	Automotive	655	81.8%	Global	871,815

a “Domestic” was defined as a company with overseas sales ratio less than 30%, and a company with more than 30% overseas sales was defined as “Global”. However, the companies indicated by ** have overseas sales ratios exceeding 30%, but because they have less than 30% of sales in each brand-bearing business, they are defined as domestic.

**Table 2:** Pearson's correlation coefficients between brand values and market capitalizations for the sample of Japanese companies.

	Total (domestic + global companies)	Domestic companies	Global companies
Correlation coefficients	0.864152^a^	0.915341^a^	0.9350639^a^

a Statistically significant with a p-value < 0.01.

**Table 3:** Cross-tabulation statistics for brand value and consolidated overseas sales ratios (global and domestic) on Japanese companies listed in Table 1.

	Brand value	
	>1 billion US dollar	<1 billion US dollar
Global	25	15
Domestic	15	25

**Table 4:** Chi-square test of independence on the cross-tabulation (Table 2) with different correction methods to measure the relationship between consolidated overseas sales ratios and brand value. Both chi-square tests indicate the relationship between consolidated overseas sales ratios and brand value are not irrelevant.

Correction methods	Chi-square	Df	p-value	Significance^a^
None	5	1	0.02535	Significant
Yates' continuity correction	4.05	1	0.04417	Significant

a Correlations with a p-value < 0.05 are considered significant.

## 2 Experimental design, materials, and methods

### 2.1 Design

Researchers can calculate the value of a company using many corporate valuation methods [1]. In this data article, we choose “brand value” based on Brand Valuation, a metric published by Interbrand Japan, Inc. (Tokyo, Japan), a branding agency.

### 2.2 Brand value and market capitalization

The brand value and consolidated overseas sales ratios for 80 Japanese companies (40 domestic and 40 global companies) for 2017 were collected from the website of Interbrand Japan Ltd [2], [3]. The sample firms' market capitalizations as of October 31, 2016 were collected from Yahoo Japan's finance website (https://finance.yahoo.co.jp). The companies with brand value, consolidated overseas sales ratio and market capitalizations were listed in Table 1.

### 2.3 Statistical analysis

We present the correlation between brand value and market capitalization in a scatter plot and in regression statistics using Pearson's correlation coefficient. Cross-tabulation statistics and Chi-square test of independence were employed to assess the correlation between brand value and consolidated overseas sales ratios of Japanese companies listed in Table 1. All statistical analyses were performed using the free R software package (available at https://www.r-project.org) [4] and Microsoft Excel (Redmond, WA, USA).
